# Supplementary material for: Novel environmental risk index reveals uneven impact of climate change on fascioliasis risk across Africa and Europe
Source: PLoS Negl Trop Dis. 2026 Oct 5;20(10):e0013821. doi: 10.1371/journal.pntd.0013821 (PMC13657299; doi:10.1371/journal.pntd.0013821)
Supplement: Supplementary file 3 [file pntd.0013821.s004.docx]

Dear editor,

We are grateful to both reviewers for their constructive comments on our manuscript. It is encouraging that both reviewers recognize the relevance and methodological strength of our study. We appreciate the opportunity to submit an improved version of the manuscript based on these comments. A detailed response to each reviewer comment is provided below, where our responses are in italics and highlighted in grey, and text that was added or changed in the manuscript is in red. Line numbers refer to the document with tracked changes.

**Reviewer #1: Methods**
- The study focuses primarily on *Galba truncatula* and *Radix natalensis* as the main intermediate host snails for *Fasciola* transmission. While these species are epidemiologically important, the exclusion of other known competent intermediate hosts may limit the completeness of the risk assessment. In particular, the potential contribution of alternative or invasive lymnaeid species under changing climatic conditions deserves further consideration. A clearer justification for restricting the analysis to these two snail species would strengthen the methodological framework.

*We* appreciate *this important point, especially given the increasing importance of* Pseudosuccinea columella*. A recent review confirmed that* G. truncatula *and* R. natalensis *are still the most important host species in Africa* (Hadebe et al., 2023)*, and* P. columella *also still plays a relatively* *minor role in Europe. We therefore chose to focus on the native hosts. We have added additional discussion of alternative host species in the method section:*

*Despite recent evidence that G. truncatula might be a cryptic species complex, we consider it as a single species because molecularly verified occurrence data is scarce [47]. Additionally, we did not include Pseudosuccinea columella, an intermediate host species for both F. hepatica and F. gigantica that is native to the Americas and is invasive in both Africa and Europe. Although we recognize the importance of this species may increase in the future, current evidence suggests that native intermediate host species remain much more important for Fasciola transmission in Africa and Europe than P. columella [48–50].*

We also further reiterated this point in the dedicated paragraph on P. columella and other alternative hosts in the discussion.
- The integration of thermal biology and SDMs is very interesting, although I would have liked a bit more detail on how uncertainty from each model component was carried into the final risk index.

Thank you for your comment. The final risk index is a product of the mean prediction of the temperature-dependent transmission capacity, while we measured performance for SDMs using 5-fold cross-validation and then fit once on all data to generate final predictions. We generated the risk index separately for each hydrological model and climate model (shown in supplementary figures U and V). Since the two components were fit with different statistical methods (Bayesian models for thermal biology and cross-validation and machine learning for SDMs), it was not feasible to propagate uncertainty from both models to the final risk index. To more clearly show the uncertainty that comes from the choice of climatic and hydrological model, we calculated various summary statistics (as suggested, see our response below) with a range of plausible values across climate and hydrological models.
- I appreciated the effort to include museum and unpublished occurrence records, especially for Africa where data are limited. Still, the strong imbalance between European and African records could potentially influence the reliability of the projections.

This is indeed a limitation, which could especially be problematic for our models of G. truncatula, which occurs on both continents but with a very limited number of occurrence records in Africa (49 records after spatial filtering). While it is impossible to completely account for sampling bias with statistical methods, we designed a background point sampling design where the number of background points is proportional to the number of occurrences in each continent (described in section 2.3.2). To further emphasize sampling bias as a limitation of this study and a need for further research, we have added two phrases:

In the methods (line 277):

“Sampling background points (sometimes known as pseudo-absences) in a way that accounts for this bias is an effective way to mitigate the effects of sampling bias in models, even though it cannot completely correct for sampling bias”

In the discussion (line 633):

“More work is needed to investigate how such variability might help Fasciola parasites and their intermediate hosts adapt to climate change in the future, especially in Africa where data on snail intermediate hosts is scarce.”

- The monthly averaging approach works well for large-scale projections, although it may smooth important seasonal transmission peaks, particularly in highly seasonal African regions.

This is a good point. To show these seasonal dynamics, we have added two supplementary figures (M and N) that show the monthly components of the risk index (temperature-based transmission capacity and hydrology) for each month. These figures clearly show seasonality across Africa (e.g. increasing risk in West Africa from June, after the start of the rainy season) as well as in Europe (higher risk in July-August during the summer months). We also added a reference to these figures in the main text:

“Temperature-dependent transmission strength in Europe is relatively low, reflecting a seasonal transmission pattern, with cessation of transmission in winter months (monthly transmission capacity shown in Fig M in S1 Supplementary Materials).”

**Reviewer #1: Results**
- The results are generally clear and well structured, and the figures do a good job of illustrating the main spatial patterns across Europe and Africa. I found the contrast between F. hepatica and F. gigantica quite convincing, especially how the temperature limits translate into very different geographic distributions. However, some of the results would benefit from a bit more quantitative summary. As it stands, a lot of the interpretation relies mainly on the maps, so it’s sometimes hard to quickly compare changes between regions or scenarios.

This is an excellent suggestion, for which we are particularly grateful. We have quantified some of the trends shown in the maps by calculating the proportion of land area where risk is increasing or decreasing for each species and on each continent, as well as the proportion of cattle and sheep held on these areas. These were also calculated separately for each climate and hydrological model to show the uncertainty associated with model choice. We refer to these numbers throughout the text, e.g.:

“Risk is also projected to decrease in Southern Europe, but to increase slightly in Northern Europe, especially in colder areas such as the British Isles, Scandinavia, and Iceland, which historically has been free of Fasciola [57], corresponding to increasing risk in 26.5% (range: 20.0-32.0%) of European land area.”

And similarly throughout the results.

- The projected increase of F. hepatica suitability in northern Europe stands out, and it would be useful to highlight more clearly how large this change is in relation to current conditions.

We agree that this is an important result of our study. The summary statistics show that the risk index for F. hepatica transmission is increasing over 26.5% (range: 20.0-32.0%) of land area, and 22.5% (range: 4.0-34.8%) of the sheep stock in Europe. We have added the revised paragraph to highlight this. For instance:

“Under climate change, most of the areas where F. hepatica transmission risk increase are areas in Scandinavia and northern Europe with modest livestock densities, with the exception of the British Isles, which is has a considerable density of sheep in particular. However, in Southern Europe, including Spain, risk is decreasing in areas with a high density of sheep. In total, most of the current stock of sheep (77.5%, range: 65.4-96.0%) and of cattle (86.9%, range: 77.0-92.5%) in Europe are held areas with declining F. hepatica transmission risk.”

We also added a supplementary map that shows the change in risk index for the end of the century compared to current conditions (Fig T), which clearly shows that the increase in risk index for F. hepatica in Scandinavia is substantial.

- The reduction in F. gigantica risk in west Africa and the Sahel is interesting, but I think the manuscript could say a bit more about the transitional areas where suitability doesn’t fully disappear but still changes noticeably.

We have revised the results section to mention the continued risk of F. gigantica transmission in the Sahel region despite declining suitability, e.g. in the last paragraph of the results:

“For F. gigantica, decreasing suitability is especially notable in the West-Africa and the Sahel region, even though transmission risk for F. gigantica will remain present”

- The overlap analysis for hybridization risk is a strong part of the results, although the choice of threshold for defining “overlap” could be better justified in the text.

The reviewer is right to point out that the 0.25 risk index threshold for both species is somewhat arbitrary. The main reason for introducing this threshold is that bivariate color maps like Fig. 5 are very hard without thresholds, but we could indeed have chosen different thresholds. To be able to show these results without thresholding, we have now added a second row of maps to the figure 5 that show the product of the risk index for both species. These map highlight areas with high environmental suitability of transmission of both species using a different metric, thus complementing the thresholded bivariate maps in Fig. 5A-C

- The livestock overlap maps are also useful, but they feel slightly secondary compared to the rest of the analysis. It might help to better connect them back to the main risk index results in the text.

We agree that these could have been more connected. We have tried to better integrate this section with the rest of the results by calculating the proportion of livestock exposed to increasing risk (as per the suggestion to use quantitative summaries above).. E.g.:

“Thus, even though F. gigantica transmission risk is increasing in only 7.8% (range: 6.7-12.2%) of African land area, these areas are home to 11.9% (range: 9.3-18.6%) of the African stock of sheep and 25.2% (range: 16.8-34.2%) of African cattle, highlighting the considerable exposure of these areas to increasing fascioliasis risk”

Uncertainty is mentioned in parts of the modelling, but it is less visible in the results section itself. A clearer presentation of variability between climate models or scenarios would make the findings more robust.

In the current version of the manuscript, we have calculated the range of plausible values of the quantitative summaries of the results. This shows that our main conclusions are very robust across all 5 climate models and 4 hydrological models (thus, 20 model combinations) tested. To make the results easier to follow, we have chosen to focus on the SSP370 scenario, but model results for both scenarios and individual models are available in the Supplementary Materials. In the revised manuscript, we have highlighted the results for the SSP126 scenario a bit more in the results. On line 389-393:

“Under the SSP126 scenario, which assumes drastic cuts to emissions, the direction of the changes is similar, risk for F. hepatica transmission is similarly projected to decline across virtually all African and most European land area. However, under this scenario the changes are more modest, and risk is projected to stabilise after the middle of the century (Figs O and T in S1 Supplementary Materials).”

And on lines 408-411 on F. gigantica, we added:

These results were robust across hydrological and climate models considered (Figs U-V in S1 Supplementary Materials), and the direction of change was consistent across the high-emission SSP370 and low-emission SSP126 scenarios (Fig O in S1 Supplementary Materials).

- Overall, the results are consistent with the modelling framework and support the main conclusions, but a bit more numerical interpretation alongside the visual outputs would strengthen the section.

Thank you for your useful comments on the results section. With the addition of estimates of the proportion of land and livestock exposed to increasing risk, we believe the results are now easier to understand.

**Reviewer #1: Discussion**
- The discussion does a good job of putting the results into context and explaining why they differ from earlier studies, especially those based on the Ollerenshaw index. It’s clear and generally well balanced. One thing that could be expanded a bit is the role of non-climatic factors, like livestock movement and local management practices, which might still strongly influence transmission even if climate suitability decreases.
We have expanded further on the importance of non-climatic factors in the dedicated paragraph (lines 531-548). Livestock movement, which we agree is a crucial factor, is now explicitly mentioned, and we state that decreasing environmental risk does not necessarily lead to a decrease in prevalence if other factors make up for it. We agree that these are important caveats to correctly understand our results.
Conclusion
- The conclusion is clear and nicely summarizes the main findings, especially the uneven effects of climate change on the two Fasciola species across regions. It could be slightly strengthened by adding a short sentence on how these results could be translated into practical surveillance or control strategies, rather than focusing only on the modelling outcomes.

Thank you for this comment. We have expanded on this aspect of our conclusion:

“Our study offers valuable insights into continental-scale dynamics under global change, expanding the evidence base for policy and showing the need to ensure surveillance and control strategies are robust to match accelerating climate change”

**Reviewer #1:** The study entitled “Novel integrated risk index reveals uneven impact of climate change on fascioliasis risk across Africa and Europe” is an interesting and original contribution, representing a valuable effort to integrate mechanistic and correlative approaches for predicting the impacts of climate change on fascioliasis transmission. The manuscript addresses an important and timely topic with clear relevance for veterinary health, epidemiology, and climate-related disease risk assessment. The modelling framework is innovative and the study is generally well-structured and comprehensive. Nevertheless, several points require clarification and further discussion before publication.
- The title is clear and interesting, although I think it feels a bit broad since the study mainly focuses on modeling environmental suitability rather than actual transmission or prevalence patterns.

We have changed the first words of the title from “novel integrated risk index” to “novel environmental risk index”, which we agree reflects the content of the study better.

- The abstract is well written overall, but I think it would be helpful to briefly mention some of the main limitations of the model, particularly that vertebrate host dynamics and local farming practices were not included. I think adding one sentence about these limitations would provide a more balanced presentation of the study and help readers better interpret the results.

Thank you for this comment. We have added a sentence stressing that these important factors are not accounted for by our risk index: “Our risk index focuses on the environmentally sensitive components of the Fasciola life cycle and thus does not account for other important factors such as local farming practices and vertebrate host densities”

- The introduction is comprehensive and gives a good overview of fascioliasis and its environmental drivers. Still, I think it would help to include a bit more context on the current burden of the disease in Africa versus Europe to better support the continental comparison made in the study.

Thank you for that comment. As a neglected disease, exact numbers of fasciolosis burden are limited and in most cases likely underestimated, and we therefore deliberately used them sparingly in the introduction. We have added a phrase at the start of the introduction to state that the actual prevalence of fascioliasis is uncertain and likely higher than the reported prevalence. We also modified the following sentence later in the introduction (line 102-104) to highlight the burden on both continents:

“This study aims to robustly predict how climate change will affect fascioliasis risk across Africa and Europe, two continents with a high burden of animal fasciolosis, as well as occasional outbreaks of human fasciolosis [4,35–37].”

- The differences between F. hepatica and F. gigantica are introduced well, but the introduction could explain more clearly why climate change is expected to affect the two species differently.

To link the differences in distribution between F. hepatica and F. gigantica more clearly to their respective climatic preferences, we have slightly re-written the opening paragraph, so it now includes the sentence:

“The two most important Fasciola species are F. hepatica and F. gigantica, of which the former is most commonly found in temperate areas, while the latter is restricted to tropical areas, reflecting the different climatic preferences of these species [8].”

- The paper describes fascioliasis as a “re-emerging” disease, but adding one or two recent epidemiological examples would make this statement stronger.

We agree with this comment and have added the following sentence to the first paragraph of the introduction: “Elsewhere, (re-)emergence has been linked to drug resistance [11], geographical spread of snail intermediate host(s) [12], movement of livestock, as well as climate change [1,13], including recent outbreaks in Egypt [14] and Italy [15]”
- In some places, the transition from the review of previous studies to the motivation for the current work feels a bit abrupt. A clearer explanation of the specific gaps this study aims to fill would improve the flow of the introduction.

We have revised the introductory paragraph in which we formulate our research gap to make the transition smoother.

**Reviewer #2**

In line 43, “….. studies, our results suggest that transmission risk will decline in across most of the range of both..”. Choose either **in** or **across** but not both.

We have corrected this typographical error, so this sentence says “…risk will decline across most of the range…”

In lines 163-164: “Thus, all parameters are dependent on temperature, except for the egg and snail death rate, which were both fixed at 0.01 in our main model formulation …”. I wonder whether the authors can elaborate why they think egg and snail death are independent of temperature. Heat stress is expected to increase the death rates of both. Do they hypothesise the effect of temperature is negligible, and if so, what could be the justification for this?

In section 2.2, I note that the authors estimate parasite transmission potential based on the number of cercariae present in the environment. However, it will be more informative if they can estimate the number of cercariae that can successfully encyst as metacercaria as many of the cercariae will die before becoming infective to the final hosts. Cercarial survival/death are also functions of the environmental temperature (and by implication climate change), which can be accounted for in the model. Without this consideration, the transmission risk is likely to be overestimated.

The reviewer raises some valid questions that we carefully considered as we designed our models. We agree that snail, egg, and cercarial survival are all strongly dependent on temperature. We chose not to fit temperature response curves for these traits for multiple reasons: 1) only few studies that met our inclusion criteria report on snail survival, and even fewer on egg and metacercarial survival (e.g. only two studies each for survival of F. hepatica and F. gigantica cercariae); 2) studies that do report often report in different ways, e.g. they report the number of snails surviving for a specific number of days, while others report median lifespan; 3) there are big differences in experimental setup – such as the conditions snails and metacercariae are exposed to; 4) studies report widely varying estimates of lifespan, e.g. Nice (1979) reports the median lifespan at 5C is 600 days, while Hodasi (1976) reports 105 days; 5) we hypothesize that experimentally measured lifespan only partly reflects real-world survival, because of additional pressure like predation, competition and strong currents. For these reasons, we chose to be conservative and fix the death rate at a single value, while keeping metacercarial encystment and survival out of the equation. Note that we performed a sensitivity analysis of this which is included in the supplementary materials (Fig K), and that all data points collected from the data are available on the repository associated with this study, even if they were not included in the analysis.

To be more transparent about why we made these modelling choices, we added a paragraph to the end of section 2.1, right after the description of the Bayesian modelling procedure:

For three other life history traits relevant to the Fasciola life cycle – the survival of snails, parasite eggs, and cercariae, we found only limited experimental evidence in the literature, which was measured and reported in varying ways. In addition, we expect that experimentally measured death rates only partly reflect real-world survival, because of additional pressure like predation and strong currents. Rather than fitting thermal response curves for these traits, we therefore fixed snail and egg death rates at 0.01 in our main model formulation and performed a sensitivity analysis (section 2 and Fig K in S1 Supplementary Materials), while keeping cercarial encystment and survival out of the estimates of the temperature-dependence of transmission.

In lines 228-229, the phrase “We set the 1 mm/day threshold following previously for modelling studies on aquatic mosquito habitats;” needs rephrasing for clarity. For instance to “We set the 1 mm/day threshold following previously for modelling studies on aquatic mosquito habitats;”

We have corrected this to “…following previous modelling studies…”

The borderline weak prediction of R. natalensis (AUC =0.69 (0.70) and TSS = 0.33 (0.32)), as indicated in Table 1, could significantly affect the prediction confidence of in the results. Could the authors discuss this more? Is it related to the limited data points available (as alluded to in the introductory section)?

The reviewer is correct to point out that these values indicate only moderately strong model performance. This could indeed be partly related to the number of data points. Given the limited amount of data, we designed our modelling strategy to avoid overfitting (e.g. by limiting the number of predictor variables and setting the regularization multiplier to 3), which somewhat reduces the ability of the model to discriminate between presence and background points (which is what both AUC and TSS measure). These scores might further be reduced by the facts that 1) R. natalensis is a generalist species found in many environments in Africa, and 2) we use a target group background approach for half the background points, thus the model is asked to discriminate between R. natalensis occurrences and occurrences of other lymnaeid species, which is probably a harder task than discriminating between occurrences and randomly drawn background points. We are nonetheless confident that the outputs of our SDMs are useful for the risk index: first, the performance statistics on training and test data are very similar, indicating that the model indeed did not overfit, and second, the resulting predicted suitability shows high suitability across most of sub-Saharan Africa, consistent with what is expected for such a widespread species. We are therefore not concerned that the SDMs would have strongly biased the final risk index. However, more data could of course further refine estimates for snail suitability. In the revised manuscript, we have added an additional call for more work on collecting data on snail intermediate hosts in Africa in particular:

“More work is needed to investigate how such variability might help Fasciola parasites and their intermediate hosts adapt to climate change in the future, especially in Africa where data on snail intermediate hosts is scarce.”

Furthermore, to make sure our SDMs capture snail suitability despite data limitations, we have added the Continuous Boyce Index (CBI), a performance measure that (unlike AUC and TSS) measures if suitability scores consistently match the ratio of presence and background points. This confirms that both models predict higher suitability in areas with more presence points, even in test datasets (scores: 0.92 for G. truncatula and 0.84 for R. natalensis models).

Fig. 4 (please edit as it is Fi. 4 currently in the caption), it would be better to support the visual displays of the change in transmission risk for Fasciola hepatica and Fasciola gigantica with statistical evidence. Are the predicted changes statistically significant? This comment applies to Fig. 5 and 6 as well.

This is a good point, similar to reviewer 1's suggestion to add quantitative summary statistics to aid interpretation of the maps in Figures 4-6. We have therefore calculated the proportion of land area and livestock exposed to increasing risk for each continent and each hydrological and climate model and reported these in the text. The reported ranges across all hydrological and climate models show that our results are robust, while the statistics aids readers in understanding the results in quantitively.

**References**

Hadebe, M. I., Manyangadze, T., Kalinda, C., Mindu, T., & Chimbari, M. J. (2023). Infection Rates of Fasciola Intermediate Host Snail Species and Their Distribution in Africa: A Systematic Review and Meta-Analysis. *Tropical Medicine and Infectious Disease*, *8*(10), Article 10. https://doi.org/10.3390/tropicalmed8100467

Hodasi, J. K. M. (1976). The effects of low temperature on Lymnaea truncatula. *Zeitschrift Für Parasitenkunde*, *48*(3), 281–286. https://doi.org/10.1007/BF00380403

Nice, N. G. (1979). *Aspects of the biology of Fasciola hepatica and its intermediate snail host Lymnaea truncatula* [PhD Thesis]. University of York.
