# Supplementary material for: Novel environmental risk index reveals uneven impact of climate change on fascioliasis risk across Africa and Europe
Source: PLoS Negl Trop Dis. 2026 Oct 5;20(10):e0013821. doi: 10.1371/journal.pntd.0013821 (PMC13657299; doi:10.1371/journal.pntd.0013821)
Supplement: Peer review history

# Peer review: Decision Letter 0

**DOI:** 10.1371/journal.pntd.0013821.r001

**Authors:** Amy Morrison

23 Jun 2026

PNTD-D-25-02168Novel integrated risk index reveals uneven impact of climate change on fascioliasis risk across Africa and EuropePLOS Neglected Tropical Diseases Dear Dr. van der Deure, Thank you for submitting your manuscript to PLOS Neglected Tropical Diseases. After careful consideration, we feel that it has merit but does not fully meet PLOS Neglected Tropical Diseases's publication criteria as it currently stands. Therefore, we invite you to submit a revised version of the manuscript that addresses the points raised during the review process. Please submit your revised manuscript by Aug 22 2026 11:59PM. If you will need more time than this to complete your revisions, please reply to this message or contact the journal office at plosntds@plos.org. When you're ready to submit your revision, log on to https://www.editorialmanager.com/pntd/ and select the 'Submissions Needing Revision' folder to locate your manuscript file. Please include the following items when submitting your revised manuscript:* A letter that responds to each point raised by the editor and reviewer(s). You should upload this letter as a separate file labeled 'Response to Reviewers'. This file does not need to include responses to any formatting updates and technical items listed in the 'Journal Requirements' section below.* A marked-up copy of your manuscript that highlights changes made to the original version. You should upload this as a separate file labeled 'Revised Manuscript with Track Changes'.* An unmarked version of your revised paper without tracked changes. You should upload this as a separate file labeled 'Manuscript'. If you would like to make changes to your financial disclosure, competing interests statement, or data availability statement, please make these updates within the submission form at the time of resubmission. Guidelines for resubmitting your figure files are available below the reviewer comments at the end of this letter. As the corresponding author, your ORCID iD is verified in the submission system and will appear in the published article. PLOS supports the use of ORCID, and we encourage all coauthors to register for an ORCID iD and use it as well. Please encourage your coauthors to verify their ORCID iD within the submission system before final acceptance, as unverified ORCID iDs will not appear in the published article. Only the individual author can complete the verification step; PLOS staff cannot verify ORCID iDs on behalf of authors.We look forward to receiving your revised manuscript. Kind regards, Amy C. Morrison, PhDSection EditorPLOS Neglected Tropical Diseases

Shaden Kamhawi

co-Editor-in-Chief

PLOS Neglected Tropical Diseases

orcid.org/0000-0003-4304-636XX

Paul Brindley

co-Editor-in-Chief

PLOS Neglected Tropical Diseases

orcid.org/0000-0003-1765-0002

**Additional Editor Comments (if provided):**     **Journal Requirements:**

If the reviewer comments include a recommendation to cite specific previously published works, please review and evaluate these publications to determine whether they are relevant and should be cited. There is no requirement to cite these works unless the editor has indicated otherwise.

1) Please ensure that the CRediT author contributions listed for every co-author are completed accurately and in full.

At this stage, the following Authors/Authors require contributions: Bryan Nyawanda. Please ensure that the full contributions of each author are acknowledged in the "Add/Edit/Remove Authors" section of our submission form.

The list of CRediT author contributions may be found here: https://journals.plos.org/plosntds/s/authorship#loc-author-contributions

2) Please upload all main figures as separate Figure files in .tif or .eps format. For more information about how to convert and format your figure files please see our guidelines:

https://journals.plos.org/plosntds/s/figures

3) Please upload a copy of Figures Fig 1, 2A-F, 3A-G, 4A-F, 5A-C, and 6A-F which you refer to in your text on pages 6, 14, 16, 18, 20, and 21. Or, if the figure is no longer to be included as part of the submission please remove all reference to it within the text.

4) Please amend your detailed Financial Disclosure statement. This is published with the article. It must therefore be completed in full sentences and contain the exact wording you wish to be published.

i) State the initials, alongside each funding source, of each author to receive each grant. For example: "This work was supported by the National Institutes of Health (####### to AM; ###### to CJ) and the National Science Foundation (###### to AM)."

ii) State what role the funders took in the study. If the funders had no role in your study, please state: "The funders had no role in study design, data collection and analysis, decision to publish, or preparation of the manuscript."

3) If any authors received a salary from any of your funders, please state which authors and which funders..

If you did not receive any funding for this study, please simply state: u201cThe authors received no specific funding for this work.u201d

**Reviewers' comments:**  Reviewer's Responses to Questions

**Key Review Criteria Required for Acceptance?**

As you describe the new analyses required for acceptance, please consider the following:

**Methods**

-Are the objectives of the study clearly articulated with a clear testable hypothesis stated?

-Is the study design appropriate to address the stated objectives?

-Is the population clearly described and appropriate for the hypothesis being tested?

-Is the sample size sufficient to ensure adequate power to address the hypothesis being tested?

-Were correct statistical analysis used to support conclusions?

-Are there concerns about ethical or regulatory requirements being met?

Reviewer #1: Methods

- The study focuses primarily on Galba truncatula and Radix natalensis as the main intermediate host snails for Fasciola transmission. While these species are epidemiologically important, the exclusion of other known competent intermediate hosts may limit the completeness of the risk assessment. In particular, the potential contribution of alternative or invasive lymnaeid species under changing climatic conditions deserves further consideration. A clearer justification for restricting the analysis to these two snail species would strengthen the methodological framework.

- The integration of thermal biology and SDMs is very interesting, although I would have liked a bit more detail on how uncertainty from each model component was carried into the final risk index.

- I appreciated the effort to include museum and unpublished occurrence records, especially for Africa where data are limited. Still, the strong imbalance between European and African records could potentially influence the reliability of the projections.

- The monthly averaging approach works well for large-scale projections, although it may smooth important seasonal transmission peaks, particularly in highly seasonal African regions.

Reviewer #2: The study fills a clear knowledge gap with clear methods

**********

**Results**

-Does the analysis presented match the analysis plan?

-Are the results clearly and completely presented?

-Are the figures (Tables, Images) of sufficient quality for clarity?

Reviewer #1: Results

- The results are generally clear and well structured, and the figures do a good job of illustrating the main spatial patterns across Europe and Africa. I found the contrast between F. hepatica and F. gigantica quite convincing, especially how the temperature limits translate into very different geographic distributions. However, some of the results would benefit from a bit more quantitative summary. As it stands, a lot of the interpretation relies mainly on the maps, so it’s sometimes hard to quickly compare changes between regions or scenarios.

- The projected increase of F. hepatica suitability in northern Europe stands out, and it would be useful to highlight more clearly how large this change is in relation to current conditions.

- The reduction in F. gigantica risk in west Africa and the Sahel is interesting, but I think the manuscript could say a bit more about the transitional areas where suitability doesn’t fully disappear but still changes noticeably.

- The overlap analysis for hybridization risk is a strong part of the results, although the choice of threshold for defining “overlap” could be better justified in the text.

- The livestock overlap maps are also useful, but they feel slightly secondary compared to the rest of the analysis. It might help to better connect them back to the main risk index results in the text. Uncertainty is mentioned in parts of the modelling, but it is less visible in the results section itself. A clearer presentation of variability between climate models or scenarios would make the findings more robust.

- Overall, the results are consistent with the modelling framework and support the main conclusions, but a bit more numerical interpretation alongside the visual outputs would strengthen the section.

Reviewer #2: Results are clear

**********

**Conclusions**

-Are the conclusions supported by the data presented?

-Are the limitations of analysis clearly described?

-Do the authors discuss how these data can be helpful to advance our understanding of the topic under study?

-Is public health relevance addressed?

Reviewer #1: Discussion

- The discussion does a good job of putting the results into context and explaining why they differ from earlier studies, especially those based on the Ollerenshaw index. It’s clear and generally well balanced. One thing that could be expanded a bit is the role of non-climatic factors, like livestock movement and local management practices, which might still strongly influence transmission even if climate suitability decreases.

Conclusion

- The conclusion is clear and nicely summarizes the main findings, especially the uneven effects of climate change on the two Fasciola species across regions. It could be slightly strengthened by adding a short sentence on how these results could be translated into practical surveillance or control strategies, rather than focusing only on the modelling outcomes.

Reviewer #2: The authors clearly discuss the results pointing the limitations of the study.

**********

**Editorial and Data Presentation Modifications?**

Use this section for editorial suggestions as well as relatively minor modifications of existing data that would enhance clarity. If the only modifications needed are minor and/or editorial, you may wish to recommend “Minor Revision” or “Accept”.

Reviewer #1: Minor revision

Reviewer #2: (No Response)

**********

**Summary and General Comments**

Use this section to provide overall comments, discuss strengths/weaknesses of the study, novelty, significance, general execution and scholarship. You may also include additional comments for the author, including concerns about dual publication, research ethics, or publication ethics. If requesting major revision, please articulate the new experiments that are needed.

Reviewer #1: The study entitled “Novel integrated risk index reveals uneven impact of climate change on fascioliasis risk across Africa and Europe” is an interesting and original contribution, representing a valuable effort to integrate mechanistic and correlative approaches for predicting the impacts of climate change on fascioliasis transmission. The manuscript addresses an important and timely topic with clear relevance for veterinary health, epidemiology, and climate-related disease risk assessment. The modelling framework is innovative and the study is generally well-structured and comprehensive. Nevertheless, several points require clarification and further discussion before publication.

- The title is clear and interesting, although I think it feels a bit broad since the study mainly focuses on modeling environmental suitability rather than actual transmission or prevalence patterns.

- The abstract is well written overall, but I think it would be helpful to briefly mention some of the main limitations of the model, particularly that vertebrate host dynamics and local farming practices were not included. I think adding one sentence about these limitations would provide a more balanced presentation of the study and help readers better interpret the results.

- The introduction is comprehensive and gives a good overview of fascioliasis and its environmental drivers. Still, I think it would help to include a bit more context on the current burden of the disease in Africa versus Europe to better support the continental comparison made in the study.

- The differences between F. hepatica and F. gigantica are introduced well, but the introduction could explain more clearly why climate change is expected to affect the two species differently.

- The paper describes fascioliasis as a “re-emerging” disease, but adding one or two recent epidemiological examples would make this statement stronger.

- In some places, the transition from the review of previous studies to the motivation for the current work feels a bit abrupt. A clearer explanation of the specific gaps this study aims to fill would improve the flow of the introduction.

Reviewer #2: I have attached the comments in a file

**********

PLOS authors have the option to publish the peer review history of their article (what does this mean?). If published, this will include your full peer review and any attached files.

If you choose “no”, your identity will remain anonymous but your review may still be made public.

**Do you want your identity to be public for this peer review?**  For information about this choice, including consent withdrawal, please see our Privacy Policy.

Reviewer #1: No

Reviewer #2: No

[NOTE: If reviewer comments were submitted as an attachment file, they will be attached to this email and accessible via the submission site. Please log into your account, locate the manuscript record, and check for the action link "View Attachments". If this link does not appear, there are no attachment files.] **Figure resubmission:**  While revising your submission, we strongly recommend that you use PLOS’s NAAS tool (https://ngplosjournals.pagemajik.ai/artanalysis) to test your figure files. NAAS can convert your figure files to the TIFF file type and meet basic requirements (such as print size, resolution), or provide you with a report on issues that do not meet our requirements and that NAAS cannot fix.

After uploading your figures to PLOS’s NAAS tool - https://ngplosjournals.pagemajik.ai/artanalysis, NAAS will process the files provided and display the results in the "Uploaded Files" section of the page as the processing is complete. If the uploaded figures meet our requirements (or NAAS is able to fix the files to meet our requirements), the figure will be marked as "fixed" above. If NAAS is unable to fix the files, a red "failed" label will appear above. When NAAS has confirmed that the figure files meet our requirements, please download the file via the download option, and include these NAAS processed figure files when submitting your revised manuscript. **Reproducibility:**  To enhance the reproducibility of your results, we recommend that authors of applicable studies deposit laboratory protocols in protocols.io, where a protocol can be assigned its own identifier (DOI) such that it can be cited independently in the future. Additionally, PLOS ONE offers an option to publish peer-reviewed clinical study protocols. Read more information on sharing protocols at https://plos.org/protocols?utm_medium=editorial-email&utm_source=authorletters&utm_campaign=protocols

Attachment Submitted filename: PNTD-D-25-02168_reviewer.pdf

# Author comment: Author response to Decision Letter 1

**DOI:** 10.1371/journal.pntd.0013821.r002

24 Aug 2026

Attachment Submitted filename: response_to_review_comments_final.docx

# Editor report: Decision Letter 1

**DOI:** 10.1371/journal.pntd.0013821.r003

**Authors:** Amy Morrison

21 Sep 2026

Dear Mr. van der Deure,

We are pleased to inform you that your manuscript 'Novel environmental risk index reveals uneven impact of climate change on fascioliasis risk across Africa and Europe' has been provisionally accepted for publication in PLOS Neglected Tropical Diseases.

Before your manuscript can be formally accepted you will need to complete some formatting changes, which you will receive in a follow up email. A member of our team will be in touch with a set of requests.

Please note that your manuscript will not be scheduled for publication until you have made the required changes, so a swift response is appreciated.

IMPORTANT: The editorial review process is now complete. PLOS will only permit corrections to spelling, formatting or significant scientific errors from this point onwards. Requests for major changes, or any which affect the scientific understanding of your work, will cause delays to the publication date of your manuscript.

Should you, your institution's press office or the journal office choose to press release your paper, you will automatically be opted out of early publication. We ask that you notify us now if you or your institution is planning to press release the article. All press must be co-ordinated with PLOS.

Thank you again for supporting Open Access publishing; we are looking forward to publishing your work in PLOS Neglected Tropical Diseases.

Best regards,

Amy C. Morrison, PhD

Section Editor

PLOS Neglected Tropical Diseases

Amy Morrison

Section Editor

PLOS Neglected Tropical Diseases

Shaden Kamhawi

co-Editor-in-Chief

PLOS Neglected Tropical Diseases

orcid.org/0000-0003-4304-636XX

Paul Brindley

co-Editor-in-Chief

PLOS Neglected Tropical Diseases

orcid.org/0000-0003-1765-0002

***********************************************************

Congratulations

# Editor report: Acceptance letter

**DOI:** 10.1371/journal.pntd.0013821.r004

**Authors:** Amy Morrison

Dear Mr. van der Deure,

We are delighted to inform you that your manuscript, "Novel environmental risk index reveals uneven impact of climate change on fascioliasis risk across Africa and Europe," has been formally accepted for publication in PLOS Neglected Tropical Diseases.

We have now passed your article onto the PLOS Production Department who will complete the rest of the publication process. All authors will receive a confirmation email upon publication.

The corresponding author will soon be receiving a typeset proof for review, to ensure errors have not been introduced during production. Please review the PDF proof of your manuscript carefully, as this is the last chance to correct any scientific or type-setting errors. Please note that major changes, or those which affect the scientific understanding of the work, will likely cause delays to the publication date of your manuscript. Note: Proofs for Front Matter articles (Editorial, Viewpoint, Symposium, Review, etc...) are generated on a different schedule and may not be made available as quickly.

Soon after your final files are uploaded, the early version of your manuscript will be published online unless you opted out of this process. The date of the early version will be your article's publication date. The final article will be published to the same URL, and all versions of the paper will be accessible to readers.

For Research Articles, you will receive an invoice from PLOS for your publication fee after your manuscript has reached the completed accept phase. If you receive an email requesting payment before acceptance or for any other service, this may be a phishing scheme. Learn how to identify phishing emails and protect your accounts at https://explore.plos.org/phishing.

Thank you again for supporting open-access publishing; we are looking forward to publishing your work in PLOS Neglected Tropical Diseases.

Best regards,

Shaden Kamhawi

co-Editor-in-Chief

PLOS Neglected Tropical Diseases

Paul Brindley

co-Editor-in-Chief

PLOS Neglected Tropical Diseases
